# The impact of erythroblast enucleation efficiency on the severity of anemia in patients with myelodysplastic syndrome

**DOI:** 10.1186/s12964-023-01353-4

**Published:** 2023-11-20

**Authors:** Chao An, Fumin Xue, Ling Sun, Haiyan Han, Yali Zhang, Yibo Hu

**Affiliations:** 1 Department of Clinical Laboratory, The Second Affiliated Hospital of Zhengzhou University, Zhengzhou, 450014 Henan China; 2 Henan Key Laboratory of Children’s Genetics and Metabolic Diseases, Children’s Hospital Affiliated to Zhengzhou University, Zhengzhou, 450000 Henan China; 3 Department of Hematology, First Affiliated Hospital of Zhengzhou University, Zhengzhou, 450052 China

**Keywords:** Enucleation, Myelodysplastic syndrome (MDS), Anemia, MAPK/ERK, PI3K/AKT

## Abstract

Anemia is the most common manifestation in myelodysplastic syndrome (MDS) patients, but the cause of ineffective hematopoiesis is not fully understood. Enucleation is an important event in the maturation process of erythroblasts. According to a series of morphological phenotypes of the pathological development of MDS erythroblasts, we speculate that there may be enucleation disorders. To verify this hypothesis, we cultured MDS bone marrow CD34^+^ cells in vitro and induced erythroblast development. The results showed that erythroblast enucleation in MDS was significantly lower than that in the normal group, and the rate of enucleation was positively correlated with hemoglobin concentration. Risk stratification of MDS was performed to further analyze the differences in enucleation among the normal group, low-middle risk group and high-risk group. The results showed that the enucleation rate of the high risk group was higher than that of the low-middle risk group but still lower than that of the normal group. Moreover, the expression of pERK and pAKT in MDS erythroblasts in the high risk group was higher than that in the normal group, while the expression of pERK and pAKT in the low-middle risk group was lower than that in the normal group. Furthermore, the enucleation of MDS was positively correlated with the phosphorylation degree of ERK and AKT. In conclusion, this study reveals that the enucleation of erythroblasts is one of the possible causes of anemia in MDS.

Video Abstract

**Supplementary Information:**

The online version contains supplementary material available at 10.1186/s12964-023-01353-4.

## Introduction

The most prominent manifestation of MDS is the significant reduction in the number of the multiple blood cell lineages in peripheral blood; this reduction is accompanied by significant morphological and developmental abnormalities, especially in erythroblasts [1, 2]. The cause of MDS anemia is often attributed to various causes of increased apoptosis in the early stage erythroid development and impaired cell differentiation in the late stage of erythroid development [3, 4]. However, it is still unknown whether there is enucleation disorder in the erythroblasts of MDS and whether the enucleation disorder is related to anemia.

In contrast to CML caused by a single gene aberration of BCR-ABL fusion [5], the pathogenesis of MDS is believed to be the result of multistep and multigene mutations [6, 7]. One of the most common genetic variations are mutations of the Ras and FLT3 genes, which lead to the continuous activation of signaling pathways that promote cell proliferation, including the MAPK/ERK and PI3K/AKT pathways [8, 9]. Another type of genetic impact is epigenetic modification abnormalities, including those for MLL, ASXL1, DNMT3A, and TET2, resulting in cell differentiation obstruction and developmental disorders [10]. Another common type of mutation is splicing factor mutations, including those for SF3B1, SRSF2, and U2AF1 [11, 12]. During the development of erythroblasts which induced in vitro, our group and other research groups found that inhibiting the activation of ERK [13] and AKT [14] significantly inhibited erythroblast enucleation. However, the expression of this pathway in MDS erythroblasts and its correlation with MDS enucleation have not been studied thus far.

According to a series of phenotypes of the pathological development of MDS erythroblasts, we speculated that the erythroblasts of MDS were likely to have an enucleation disorder during maturation. To verify our speculation, the bone marrow of newly treated MDS patients and normal adults was collected to induce erythroblast differentiation in vitro, and flow cytometry was used to detect the rate of enucleation between the MDS and normal groups. This study is the first to observe the abnormal development of MDS erythroblasts from the perspective of enucleation, and the establishment of relevant experimental methods will enable us to further understand the cause and mechanism of MDS anemia.

## Materials and methods

### Experimental material

Bone marrow from 53 patients with initially treated MDS was obtained from the Department of Hematology of the First Affiliated Hospital of Zhengzhou University, China. All patients met the “minimum diagnostic criteria for MDS in Vienna 2006”. Exclusion criteria were as follows: patients with other blood diseases, kidney disease, liver disease, immune disease and those who were uncooperative. There were 28 males and 25 females in the patient group. The median age was 54 years (21–77), and the average initial hemoglobin concentration was 69 g/L (28–136). There were 10 patients with RA (18.8%), 3 patients with RARS (5.6%), 14 patients with RCMD (26.4%), 12 patients with RAEB-1 (22.6%), 8 patients with RAEB-2 (15.09%), and 6 patients with MDS-U (11.3%). According to the standard IPSS scores, 43 patients were at medium–low risk, and 10 were at high-risk. A total of 17 normal adult bone marrow samples (donors of bone marrow for transplant) were included in the control group. This group included 9 males and 8 females, with a median age of 43 years (18–58). All of MDS patients and normal donors had given informed consent in accordance with institutional guidelines.

## Detection method

### Purification and culture of CD34^+^ cells

Bone marrow was collected from MDS patients and healthy individuals to extract CD34^+^ cells, which were selected by a magnetic-activated cell sorting system (Miltenyi Biotec., USA). Then, CD34^+^ cells were cultured in vitro using a three-stage erythroblast induction system. The cell culture protocols used have been previously described [15]. The cells number were counted every two days.

### Flow cytometry analysis

Enucleation was analyzed with the nucleic acid dye Hoechst (ThermoFisher, MA). Flow cytometry analysis was performed using Flowjo software.

### Western blot analysis

Whole erythroblasts were lysed with RIPA buffer (Millipore, MA) added with protease inhibitor (Sigma-Aldrich) and phosphatase inhibition cocktails (Roche, Basel, Switzerland). Anti-pERK, anti-ERK, anti-pAKT and anti-AKT antibodies were from Cell Signaling Technology. Western blot analysis was performed as described previously [13].

### Cytospin preparation

A total of 1 × 10^5^ cells in 200 µL were cytospun onto coated slides using Thermo Scientific Shandon Cytospin. The slides were stained with May-Grünwald (Sigma MG500) solution for 5 min, rinsed in 40 mM Tris buffer (pH 7.2) for 90 s, and subsequently stained with Giemsa solution (Sigma GS500) for 15 min. The cells were imaged using a Leica DM2000 inverted microscope.

### Analysis of data

The experimental data were independently repeated three times. Correlation analysis was conducted using the Pearson method. Statistical analysis of differences between two or more groups was performed using GraphPad Prism software. A *p* value < 0.05 was considered to indicate statistical significance.

## Results

### The enucleation efficiency of bone marrow erythroblasts from the MDS group that were cultured in vitro was lower than that from the normal group

Erythroblast differentiation was induced in vitro by the three-stage erythroid culture system in both the MDS patient group and the normal group [15]. In this culture system, a small number of cells in the normal group began to undergo enucleation from day11. Then, the enucleation gradually increased and reached the highest rate at day15. After that, a relatively high enucleation rate was maintained for a long period of time. According to our experimental data, there is a certain degree of delay in the differentiation of erythroblasts in MDS. Usually, the enucleation rate is highest at day17, and then the enucleation rate remains at this higher stage, forming a plateau. Therefore, we tested the enucleation rate of the normal group and MDS group at day17. At this time, the enucleation rate of erythroblasts in both the MDS and normal groups was maintained at the highest stage.

**Fig. 1:**
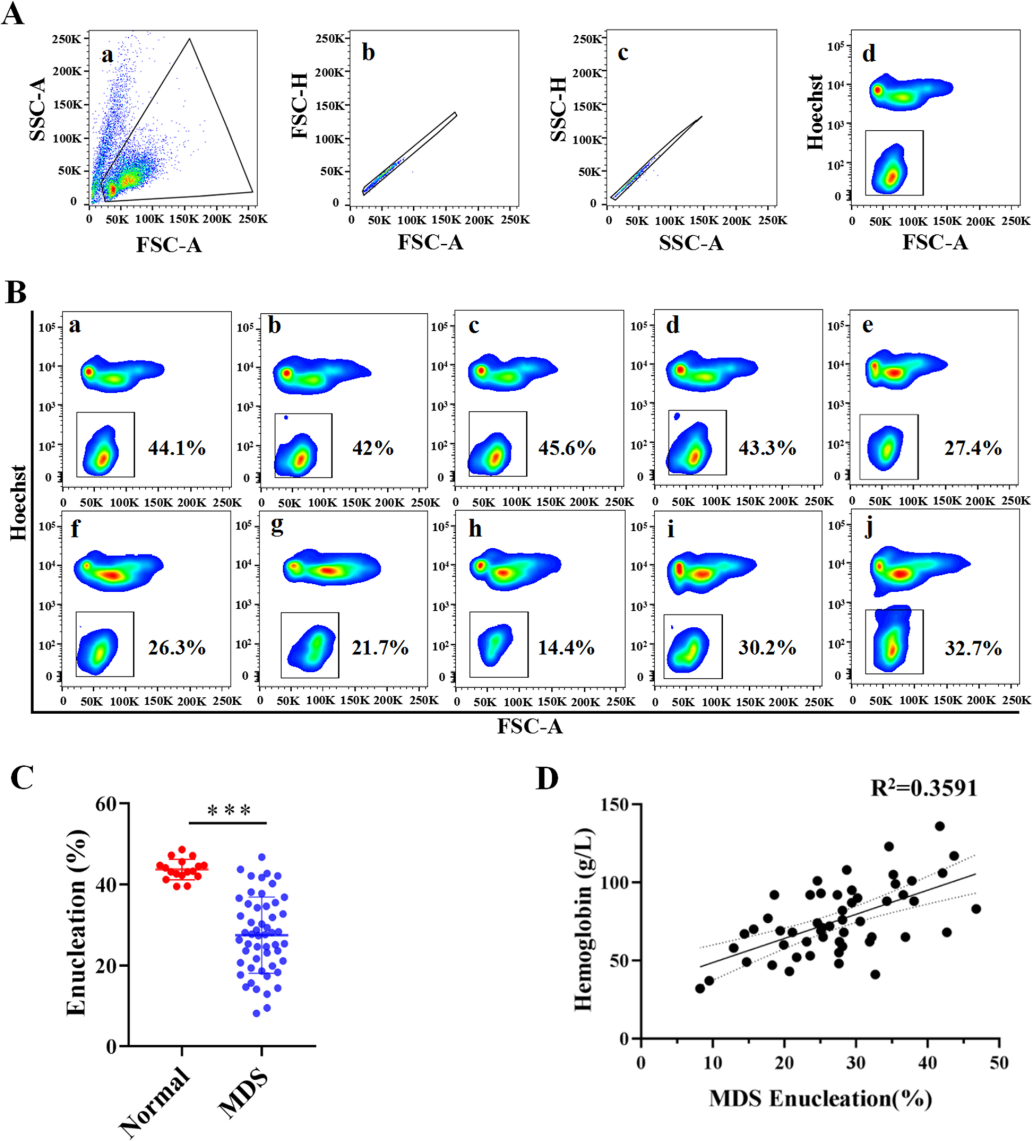
The enucleation of erythroblasts in the MDS group was lower than that in the normal group. **A** Gating strategy for erythroblast enucleation. (A:a) All events are displayed on this plot of forward scatter area vs side scatter area, exclusion of dead cells and debris. (A:b-c) Exclusion of doublets on a plot of forward scatter area vs height and side scatter area vs height. (A:d) Singles are displayed. Hoechst negative cells are gated, these are mature red blood cells. **B** Flow cytometry detection of erythroblast enucleation in normal control individuals and patients with different subtypes of MDS. (B: a-d) Normal group of erythroblast enucleation. (B: e-j) Erythroblast enucleation in the MDS subtype was RA, RARS, RCMD, RAEB-1, RAEB-2 and MDS-U. **C** Quantitative analysis statistics of the rate of enucleation in the MDS and normal groups. **D** Pearson correlation analysis showed a positive correlation between enucleation and hemoglobin concentration in MDS patients (*r* = 0.5992, *p* < 0.01). The results are expressed as the mean ± SD, and * represents *p* < 0.05, ** represents *p* < 0.01, and *** represents *p* < 0.001

The results showed that 2 cases of MDS patients had a enucleation rate lower than 10%, 11 cases had a rate of 10–20%, 20 cases had a rate of 20–30%, 13 cases had a rate of 30–40%, and only 7 cases had a rate higher than 40%. Additionally, the rate of enucleation in the 17 samples from the normal group was 38–48%. The typical results of the enucleation rate of each subtype of the MDS and normal groups are shown in Fig. 1A. Compared with that in the normal control group, the rate of enucleation in the MDS group was significantly decreased (Fig. 1B). Meanwhile, We also examined the difference between MDS and normal group on cells growth. The growth curve shown in Supplementary Fig. 1 demonstrate that erythroid cells growth in MDS group was significantly lower than that in normal group. Furthermore, Pearson correlation analysis was conducted between the rate of enucleation and the hemoglobin concentration of MDS patients at initial diagnosis. The results showed that the rate of MDS enucleation was positively correlated with the hemoglobin concentration (Fig. 1C).

### The enucleation efficiency of erythroblasts and the expression levels of pERK and pAKT were significantly different in MDS patients with different prognosis stratifications

**Fig. 2:**
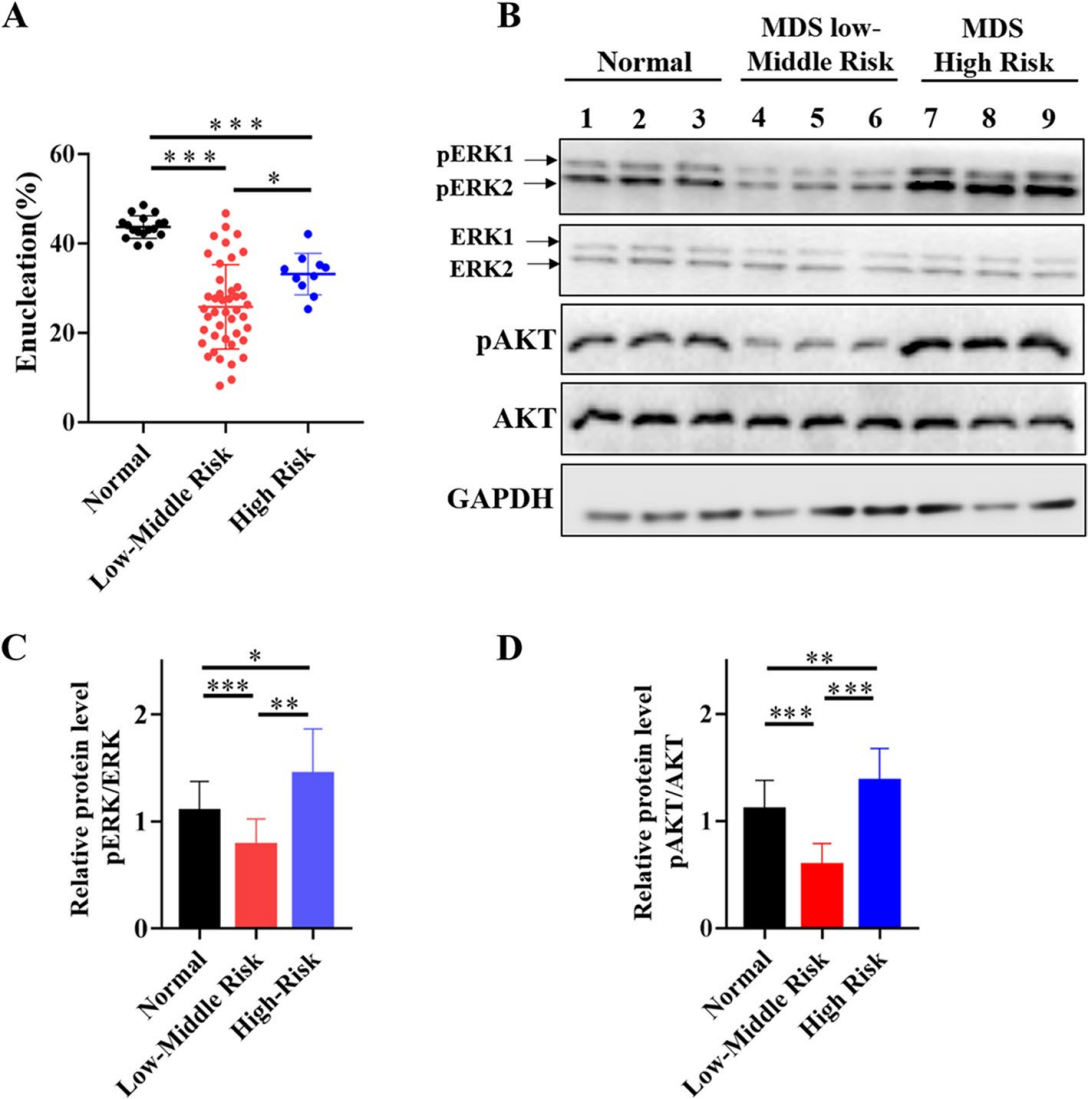
The enucleation efficiency of erythroblasts and the expression levels of pERK and pAKT were significantly different in MDS patients with different prognosis stratifications. **A** Quantitative analysis statistics of erythroblast enucleation in the normal group, low-middle risk MDS group and high risk MDS group. The expression levels of pERK and pAKT in erythroblasts in the high risk MDS group were higher than those in the normal and low-middle risk MDS groups, and the expression levels of pERK and pAKT in the low-middle risk MDS group were lower than those in the normal and high risk MDS groups. **B** Western blotting was used to detect the expression levels of pERK and pAKT in day17 erythroblasts from MDS patients and the normal group. GAPDH served as the loading control. **C** Quantitative analysis of pERK protein expression statistics. **D** Quantitative analysis of pAKT protein expression statistics. The results are expressed as the mean ± SD and * represents* p* < 0.05, ** represents *p* < 0.01, and *** represents *p* < 0.001

The International Prognostic Score System (IPSS) evaluates risk based on a patient's karyotype, the proportion of blasts in the bone marrow, and the degree of hemocytopenia [16]. High risk patients have more karyotype changes (gene mutations) or more blasts than low-middle risk patients. To determine whether the rate of enucleation of MDS in different stratifications was different, risk stratification of MDS was performed to further compare the rate of enucleation among all groups. The results showed that the rate of enucleation of MDS in the high risk group and low-middle risk group was significantly lower than that in the normal group, but the enucleation of MDS in the high risk group was significantly higher than that in the low-middle risk group (Fig. 2A). These results suggest that MDS patients with different risk levels may differ in the biological behavior of erythroblast enucleation due to differences in chromosome or gene mutations in hematopoietic stem cells. In view of the important role of the MAPKs/ERK and PI3K/AKT pathways in the occurrence of hematologic malignant diseases, we further detected the changes in pERK and pAKT of erythroblasts at day17 between individuals in the normal group and MDS patients with different stratifications. Typical western blotting results of pERK and pAKT are shown in Fig. 2B. The expression levels of pERK and pAKT in the high risk group was significantly higher than those in the normal group and the low-middle risk group, and the expression levels of of pERK and pAKT in the low-middle risk group was lower than those in the normal group and the high risk group (Fig. 2C and D).

### Correlation analysis of erythroblast enucleation and the phosphorylation of ERK and AKT in MDS patients

**Fig. 3:**
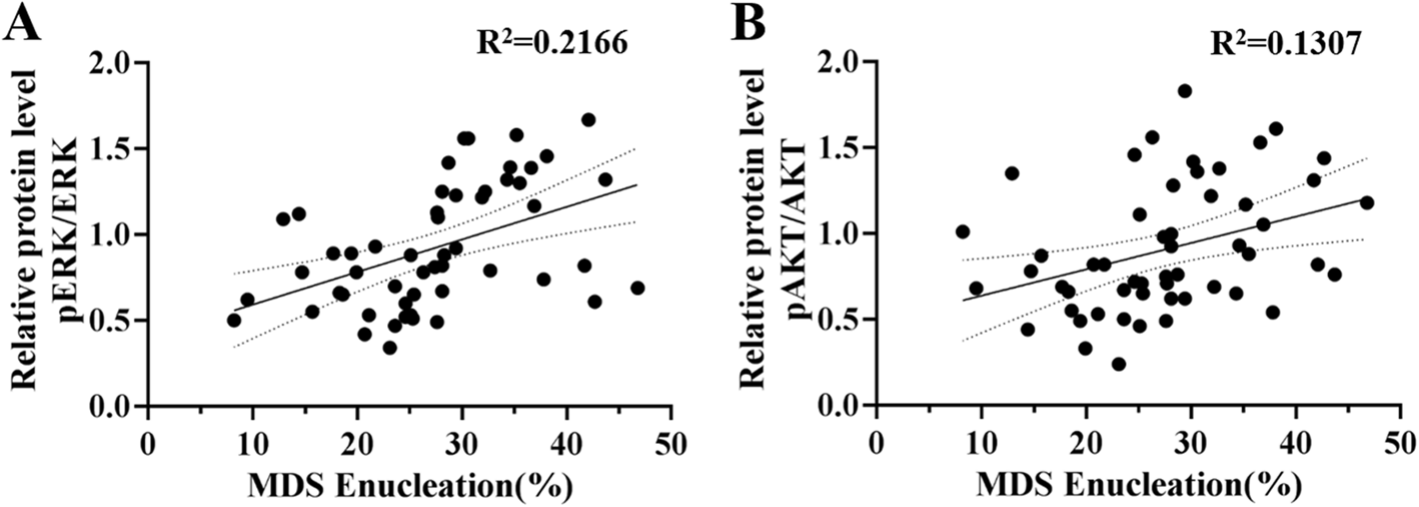
Pearson correlation analysis of the erythroblasts enucleation rate of MDS and phosphorylation of ERK and AKT. **A** The enucleation rate of MDS patients was positively correlated with the degree of ERK phosphorylation (*r* = 0.4654, *p* < 0.01). **B** The rate of enucleation in MDS patients was positively correlated with the phosphorylation of AKT (*r* = 0.3615, *p* < 0.01)

We have previously found that the dephosphorylation of ERK and AKT can inhibit the enucleation of erythroblasts [13, 14]. The results of the upper section showed that the activation degrees of ERK and AKT in MDS erythroblasts with different risk levels were different, and the enucleation was different. These results suggest that the phosphorylation degree of ERK and AKT in MDS patients may be one of the factors affecting their enucleation. Pearson correlation analysis was performed to verify the relationship between the enucleation of MDS and the phosphorylation degree of erythroblast ERK and AKT. The results showed that the enucleation efficiency of MDS was weakly positively correlated with the phosphorylation of ERK (*r* = 0.4654, *p* < 0.01) and AKT (*r* = 0.3615, *p* < 0.01) (Fig. 3A and B).

### Morphological analysis of erythroblasts from MDS patients cultured in vitro

**Fig. 4:**
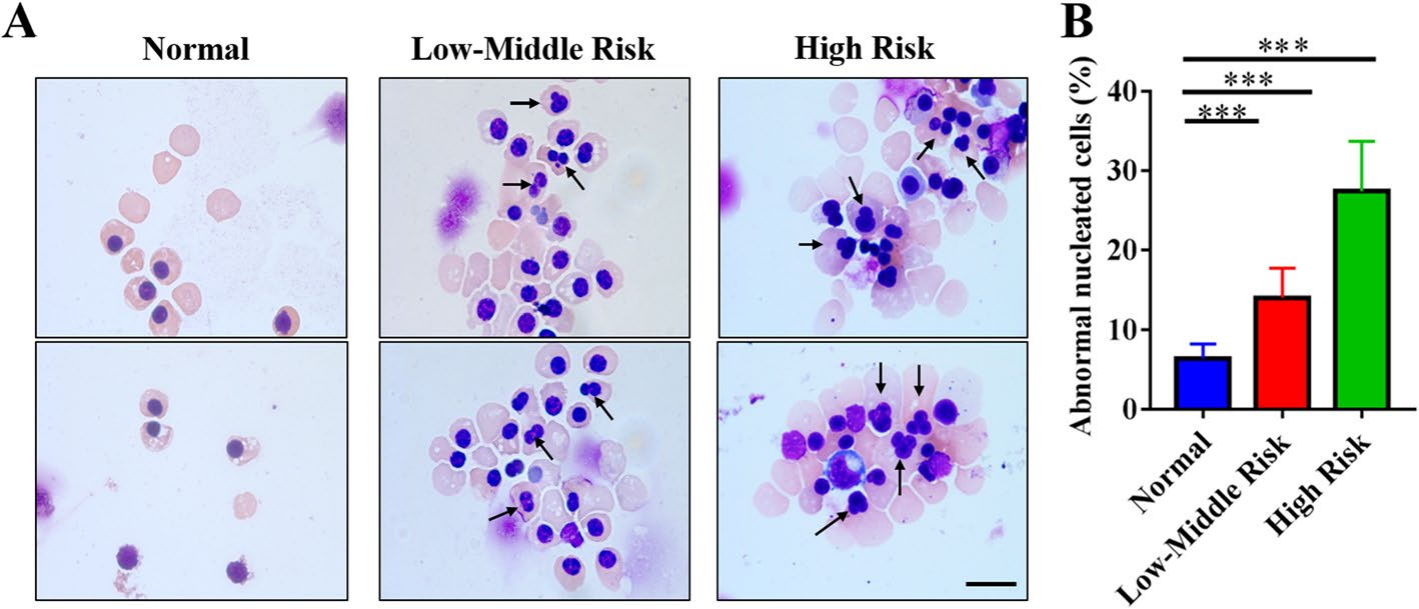
The abnormality rate of MDS erythroblast nuclei in the high risk group was higher than that in the normal group and low-middle risk group. **A** The morphology of erythroblasts from MDS patients with different risk stratifications cultured in vitro (the black arrow indicates malformed nucleus), with scale bar = 10 μm. **B** Quantitative analysis of the enucleation efficiency of erythroblasts from MDS patients with different risk stratifications. Nuclear malformations include binucleation, nuclear budding, multinucleation, and megaloblastosis, 100 cells of each group from three independent experiments were used for the quantification. The results are all expressed as the mean ± SD; * indicates *p* < 0.05,** indicates *p* < 0.01,*** indicates *P* < 0.001

Morphological changes in orthochromatic erythroblasts from the normal group, low-middle risk group and high risk group were further observed. The results showed that the frequency of nuclear malformation in the high risk group was approximately 30%, which was significantly higher than that in the low-middle risk group (15%) and normal group (5%) (Fig. 4B). Nuclear malformations often manifest as binucleation, nuclear budding, multinucleation, and megaloblastosis (Fig. 4A). These findings suggest that although orthochromatic erythroblast pERK and pAKT expression is increased in high risk MDS, there may be more chromosome malformations or gene mutations that could lead to abnormal cell division, resulting in increased nuclear malformations. These inhibiting factors offset the promoting effect of ERK and AKT phosphorylation on the enucleation of erythroblasts. The rate of enucleation was still lower than that of the normal group.

## Discussion

Most previous studies on MDS anemia have focused on apoptosis and developmental abnormalities in erythroblasts [17–19]. However, in this study, we first explored pathological development in MDS erythroblast enucleation, revealing that a great number of MDS patients exhibit abnormal enucleation rates that are positively correlated with hemoglobin concentration.

With the increasing understanding of normal erythroblast development, abnormal manifestations at different stages of erythroblast differentiation can be accurately quantified based on changes in surface markers. For instance, the ratio of Pro-E: EB-E: LB-E: Poly-E: Ortho-E cells during normal erythroblast differentiation in later stages is 1:2:4:8:16; however, this ratio does not apply to MDS patients [15]. In fact, the entire process of erythropoiesis under physiological conditions is a result of the interaction between erythroblasts and macrophages on the erythroblastic island [20, 21]. In this study, hematopoietic stem cells from the bone marrow of MDS patients were cultured in vitro to exclude any influence from the hematopoietic microenvironment. The MDS erythroblasts showed reduced proliferation, abnormal development and disorder in enucleation, and these abnormal erythroblasts were eventually phagocytosed by macrophages. Therefore, enucleation disorder further exacerbates the ineffective hematopoietic function of MDS. Our findings also support the notion that the enucleation rate in MDS patients is positively correlated with their hemoglobin concentration.

Our previous research demonstrated that the MAPK/ERK pathway modulates erythroblast enucleation by regulating vesicle formation [13]. In this study, we observed a decrease in pERK and pAKT expression levels in MDS patients categorized as low-middle risk, and high risk patients exhibited an increase in both pERK and pAKT expression levels. These findings are consistent with a previous report [22–24]. Our results suggest that apoptosis is the primary cause of abnormal erythroblast development in low-middle risk patients. However, in high risk patients, apoptosis may be blocked, and hyperproliferation or increased risk of leukemia transformation due to disease progression or the accumulation of related gene mutations can occur. We observed a positive correlation between erythroblast enucleation and ERK and AKT phosphorylation in MDS patients. However, while pERK and pAKT expression was significantly higher in high risk MDS patients than in the normal group, their enucleation was lower than that of the normal group. This paradoxical outcome can be accounted for by the accumulation of additional chromosome abnormalities or gene mutations in high risk MDS patients. Despite the activation of the ERK and AKT pathways, their positive impact on erythroblast enucleation is counteracted by mutations in other genes, such as increased nuclear malformations or cytoskeletal abnormalities [25, 26].

## Conclusion

In summary, abnormality of erythroblast enucleation is a prevalent feature in the majority of MDS patients. The pathogenesis underlying anemia is not only attributed to the increased apoptosis of early erythroid progenitor cells and impaired differentiation of late erythroblasts but also inefficient enucleation considered one of the potential causes of MDS-associated anemia.

### Supplementary Information

**Additional file 1: Supplementary Figure 1.** Growth curve of normal group and MDS group CD34^+^ cells. The results are all expressed as the mean±SD; * indicates *p*<0.05,** indicates *p*<0.01,*** indicates *P*<0.001.

## Data Availability

The datasets used and/or analysed during the current study are available from the corresponding author upon request. All data generated or analysed during this study are included in this published article.

## Abbreviations

MDS: Myelodysplastic syndrome
CML: Chronic Myeloid Leukemia
D: Day
IPSS: The International Prognostic Score System
Pro-E: Proerythroblasts
EB-E: Early basophilic erythroblasts
LB-E: Late basophilic erythroblasts
Poly-E: Polychromatic erythroblasts
Ortho-E: Orthochromatic erythroblasts
