# Supplementary material for: The impact of erythroblast enucleation efficiency on the severity of anemia in patients with myelodysplastic syndrome
Source: Cell Commun Signal. 2023 Nov 20;21:332. doi: 10.1186/s12964-023-01353-4 (PMC10658927; doi:10.1186/s12964-023-01353-4)

**Figure Legends**

Supplementary Figure 1. Growth curve of normal group and MDS group CD34^+^ cells. The results are all expressed as the mean±SD; * indicates *p*<0.05,** indicates *p*<0.01,*** indicates *P*<0.001.


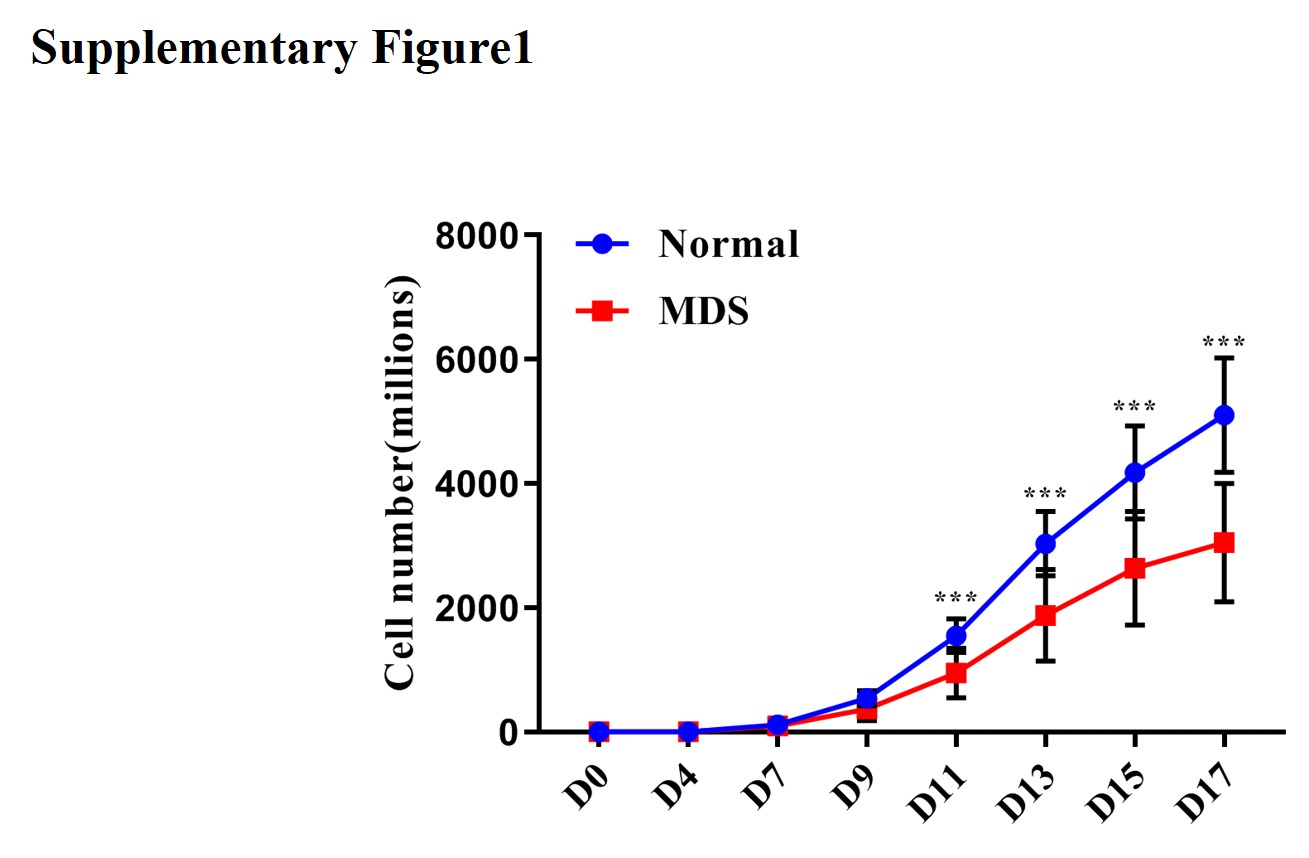

Supplement: Supplementary file 2 — Additional file 1: Supplementary Figure 1. Growth curve of normal group and MDS group CD34+ cells. The results are all expressed as the mean±SD; * indicates p<0.05,** indicates p<0.01,*** indicates P<0.001. [file 12964_2023_1353_MOESM1_ESM.doc]
